# Big Five Traits and Facets, Internet Gaming Disorder, and Social Media Addiction

**DOI:** 10.1177/00207640251400795

**Published:** 2025-12-26

**Authors:** Peter K. H. Chew, Ashleigh Hazra, Ian C. L. Quek, Shamus H. M. Seng, N. Vickneshwaran, Chelsia W. X. Tan

**Affiliations:** 1 James Cook University, Singapore, Singapore

**Keywords:** big five, traits, facets, internet gaming disorder, social media addiction

## Abstract

**Background:**

While many studies have examined the relationships between the Big Five traits, and Internet Gaming Disorder (IGD) and Social Media Addiction (SMA), limited studies have considered the Big Five facets (i.e., narrowly defined traits).

**Aims:**

The current study aims to contribute to the literature by examining the relationships between the Big Five traits and facets, and IGD and SMA among adults in the general population.

**Method:**

Participants were a convenience sample of 246 gamers and/or social media users. Their age ranged from 18 to 88 (*M* = 25.21, *SD* = 8.38). They completed instruments that assess the Big Five traits and facets, IGD, and SMA.

**Results:**

At the trait level, the results showed that conscientiousness and negative emotionality were protective and risk factors for IGD and SMA, respectively. Furthermore, agreeableness was a protective factor for IGD whereas extraversion was a risk factor for SMA. At the facet level, trust, respectfulness, and responsibility were protective factors for IGD whereas emotional volatility and depression were the main risk factors for SMA.

**Conclusions:**

The results highlighted the advantages of using facets as predictors and have implications for both research and clinical practice. Limitations include the unreliability of two facets and the cross-sectional design of the study. Future research directions include using better instruments to assess the Big Five traits and facets and conducting longitudinal studies to assess the direction of causality.

## Introduction

Research that has examined the relationships between the Big Five traits, and Internet Gaming Disorder (IGD) and Social Media Addiction (SMA) has yielded mixed results. While meta-analytic studies have clarified those results by exploring moderator variables like age and country (Chew, 2022; Huang, 2022), the results could be further elaborated by considering the Big Five facets (i.e., narrowly defined traits). Unfortunately, it appears that only one study has pursued this line of research among adolescents at risk for IGD and SMA (Wartberg et al., 2023). The current study aims to contribute to the literature by examining the relationships between the Big Five traits and facets, and IGD and SMA among adults in the general population.

### IGD and SMA

IGD is defined as “a pattern of excessive and prolonged Internet gaming that results in a cluster of cognitive and behavioral symptoms, including progressive loss of control over gaming, tolerance, and withdrawal symptoms, analogous to the symptoms of substance use disorders” and included in the *Diagnostic and Statistical Manual of Mental Disorders*, 5th edition (DSM-5) as a condition that warrants further research (American Psychiatric Association, 2013, p. 796). According to the DSM-5, individuals who meet five or more of the following criteria during the past 12 months would meet the diagnostic criteria for IGD: (1) preoccupation with gaming, (2) withdrawal symptoms, (3) tolerance, (4) unsuccessful attempts to reduce or stop gaming, (5) loss of interest in other activities because of gaming, (6) continued gaming despite problems, (7) deceiving family members or others about amount of gaming, (8) gaming to relive negative moods, and (9) risk or loss of a relationship, job, or educational or career opportunity because of gaming.

Similarly, SMA is defined as uncontrollable and excessive use of social media, leading to impairment in important life domains like work and relationships (Andreassen et al., 2016). However, unlike IGD, SMA has not been included in the DSM-5 and does not have an official set of diagnostic criteria. Instead, the components model of addiction is often used to assess SMA (Griffiths, 2005). According to the model, the six criteria for addiction are: (1) salience, (2) mood modification, (3) tolerance, (4) withdrawal, (5) conflict, and (6) relapse. These criteria distinguish between healthy and problematic use of social media.

Both IGD and SMA are associated with a range of negative consequences. For example, students with IGD or SMA tend to have lower academic achievement (Hawi et al., 2018; Hou et al., 2019). More generally, individuals with IGD or SMA tend to report negative emotional states like depression, anxiety, and stress (Tan & Chew, 2024), and poorer sleep quality (Krishnan & Chew, 2024). Given these consequences, research has been conducted to identify risk factors for IGD and SMA.

The Interaction of Person-Affect-Cognition-Execution (I-PACE) model is often used as a framework to study risk factors for specific internet-use disorders like IGD and SMA (Young & Brand, 2017). According to the model, a person’s core characteristics (e.g., personality) interact with a range of cognitive (e.g., coping style) and affective (e.g., craving) variables, leading to the development or maintenance of specific internet-use disorders. One core characteristic often studied as a risk factor is the Big Five traits.

The Big Five traits, consisting of open-mindedness (O), conscientiousness (C), extraversion (E), agreeableness (A), and negative emotionality (N), summarize individual differences in affect, behavior, and cognition (Soto & John, 2017). Traits are broadly defined and they can predict a wide range of criteria. Currently, research has yielded mixed results with regards to the relationships between the Big Five traits, and IGD and SMA. For example, while one study found a nonsignificant relationship between agreeableness and IGD (Dieris-Hirche et al., 2020), another study found a significant negative relationship between the variables (Ok, 2021). Consequently, meta-analyses have since been conducted to synthesize and clarify the results (Chew, 2022; Huang, 2022). For IGD, a meta-analysis of 12 studies showed that it was negatively correlated with conscientiousness, extraversion, and agreeableness, and positively correlated with negative emotionality (Chew, 2022). For SMA, a meta-analysis of 63 studies showed that it was negatively correlated with conscientiousness and agreeableness, and positively correlated with negative emotionality (Huang, 2022). Overall, the meta-analyses have clarified and found potential reasons (e.g., age and country) for the mixed results. However, the results could be further elaborated by considering the Big Five facets.

The Big Five traits and facets are conceptualized as a hierarchical model, where facets are subsumed under traits, and items are used to assess both traits and facets concurrently (Costa & McCrae, 1995). An example of this model as used by the Big Five Inventory 2 is shown in Table 1 (Soto & John, 2017). In contrast to traits, facets are narrowly defined and can only predict a restricted range of criteria, albeit with increased accuracy. The use of facets as predictors could increase our understanding of the risk factors of IGD and SMA. For example, negative emotionality is a risk factor for both IGD (Chew, 2022) and SMA (Huang, 2022). The use of facets enables researchers and clinicians to identify the specific aspects of negative emotionality (i.e., anxiety, depression, and/or emotional volatility) that contributes to IGD and SMA.

**Table 1.** The Big Five Hierarchical Model as Used by the Big Five Inventory 2.

Traits and facets	Sample items
Open-mindedness	
Intellectual curiosity	Is curious about many different things
Aesthetic sensitivity	Is fascinated by art, music, or literature
Creative imagination	Is original, comes up with new ideas
Conscientiousness	
Organization	Is systematic, likes to keep things in order
Productiveness	Is efficient, gets things done
Responsibility	Is dependable, steady
Extraversion	
Sociability	Is outgoing, sociable
Energy level	Is full of energy
Assertiveness	Has an assertive personality
Agreeableness	
Compassion	Is compassionate, has a soft heart
Respectfulness	Is respectful, treats others with respect
Trust	Has a forgiving nature
Negative emotionality	
Anxiety	Can be tense
Depression	Often feels sad
Emotional volatility	Is moody, has up and down mood swings

Currently, it appears that only one study has examined the relationships between the Big Five facets, and IGD and SMA (Wartberg et al., 2023). Bivariate analyses showed that IGD was significantly correlated with all the facets except for assertiveness (E) whereas SMA was significantly correlated with all the facets except for aesthetic sensitivity (O), sociability (E), and assertiveness (E). Subsequently, multivariate analyses showed that aesthetic sensitivity (O), organization (C), productiveness (C), assertiveness (E), and anxiety (N) significantly predicted IGD whereas only anxiety (N) significantly predicted SMA. However, the generalizability of the results was limited because the study recruited adolescents (mean age = 16.83 years) at risk for IGD and SMA.

The current study aimed to address the limitation of the previous study and extend on their results (Wartberg et al., 2023) by examining the relationships between the Big Five traits and facets, and IGD and SMA among adults (mean age = 18 years and above) in the general population. With regards to traits, consistent with previous meta-analyses (Chew, 2022; Huang, 2022), it was hypothesized that IGD would be negatively correlated with conscientiousness, extraversion, and agreeableness, and positively correlated with negative emotionality. It was also hypothesized that SMA would be negatively correlated with conscientiousness and agreeableness, and positively correlated with negative emotionality. With regards to facets, given the limited research in this area (Wartberg et al., 2023), the current study adopted an exploratory approach and no hypotheses were postulated.

## Method

### Participants

Participants were a convenience internet sample of 347 gamers and/or social media users recruited from online gaming/social media platforms, the university’s research participation pool, and via word-of-mouth. They were at least 18 years old and were not diagnosed with any psychological disorders. A total of 101 participants were removed via listwise deletion from the dataset due to substantial missing data (i.e., no responses on all items of one or more instruments) and multivariate outliers (i.e., Mahalanobis distance exceeding the critical value where alpha level = .001), resulting in a final sample of 246 participants (61.4% females, 35.8% males, 1.6% non-binary, and 1.2% prefer not to say). Their age ranged from 18 to 88 (*M* = 25.21, *SD* = 8.38). The sample size exceeded the minimum required sample size of 119 (104 + m, where m = number of predictors) for multiple regression analysis (Green, 1991).

### Instruments

#### The Big Five Inventory 2

The Big Five Inventory 2 is a 60-item instrument designed to assess the Big Five traits and facets (see Table 1) (Soto & John, 2017). Responses were made on a 5-point Likert Scale that ranges from 1 = *Disagree Strongly* to 5 = *Agree Strongly*. Negatively worded items were reverse-scored and the relevant item scores were summed for each trait and facet, with higher scores indicating higher levels of the respective trait and facet. Each trait was assessed using 12 items and the scores ranged from 12 to 60 whereas each facet was assessed using 4 items and the scores ranged from 4 to 20. The factor structure of the instrument has been supported by exploratory and confirmatory factor analyses (Soto & John, 2017). The Cronbach’s alpha for the traits and facets ranged from .83 to .90 and .66 to .85, respectively.

#### The Internet Gaming Disorder Scale Short Form

The Internet Gaming Disorder Scale Short Form is a 9-item instrument designed to assess IGD based on the DSM-5 criteria (Pontes & Griffiths, 2015). Responses were made on a 5-point Likert Scale that ranges from 1 = *Never* to 5 = *Very Often*. The item scores were summed, with higher scores indicating higher levels of IGD. Scores for the instrument ranged from 9 to 45. In addition, responses given as a 4 or 5 (Often or Very Often) were coded as an endorsement of the criterion. An endorsement of five or more criteria is indicative of IGD (American Psychiatric Association, 2013). The factor structure of the instrument has been supported by exploratory and confirmatory factor analyses (Chew et al., 2025; Pontes & Griffiths, 2015). The Cronbach’s alpha for the instrument was .87.

#### The Bergen Social Media Addiction Scale

The Bergen Social Media Addiction Scale is a 6-item instrument designed to assess SMA based on the components model of addiction (Andreassen et al., 2012, 2016). Responses were made on a 5-point Likert Scale that ranges from 1 = *Very Rarely* to 5 = *Very Often*. The item scores were summed, with higher scores indicating higher levels of SMA. Scores for the instrument ranged from 6 to 30. In addition, responses given as a 4 or 5 (Often or Very Often) were coded as an endorsement of the criterion. An endorsement of all six criteria is indicative of SMA (i.e., a strict monothetic classification) (Cheng et al., 2021). The factor structure of the instrument has been supported by a confirmatory factor analysis (Andreassen et al., 2012). The Cronbach’s alpha for the instrument was .83.

### Procedure

The study was conducted using Qualtrics, an online survey platform. Upon providing informed consent, participants completed a demographic form that asked for their age and gender. Subsequently, they completed the Big Five Inventory 2 (Soto & John, 2017), the Internet Gaming Disorder Scale Short Form (Pontes & Griffiths, 2015), and the Bergen Social Media Addiction Scale (Andreassen et al., 2016). These instruments were administered in English and in a randomized order to control for fatigue and order effects. Eligible participants received course credits for their time. The study was conducted according to the guidelines of the Declaration of Helsinki and approved by the university’s Human Research Ethics Committee (approval number: H9443).

## Results

The data was analyzed using IBM SPSS Statistics Version 21 with the alpha level set at .05. A total of 23 participants (9.3%) met the DSM-5 IGD criteria whereas 5 participants (2.0%) met the strict monothetic classification for SMA. Preliminary analyses were conducted to ensure no violation of the assumptions of normality, linearity, homoscedasticity, multicollinearity, and independence of errors. Furthermore, multivariate outliers were removed before analyses.

### The Big Five Traits

A series of Pearson correlations were conducted to examine the relationships between the Big Five traits and IGD and SMA. The results are presented in Table 2. Conscientiousness, extraversion, and agreeableness were negatively correlated with IGD whereas negative emotionality was positively correlated with IGD. Conscientiousness and agreeableness were negatively correlated with SMA whereas negative emotionality was positively correlated with SMA.

**Table 2.** Descriptives and Pearson Correlations of the Big Five Traits, Internet Gaming Disorder (IGD), and Social Media Addiction (SMA).

Variables	1	2	3	4	5	6	7
1. Open-mindedness	–						
2. Conscientiousness	−.07	–					
3. Extraversion	.24***	.23***	–				
4. Agreeableness	−.02	.61***	.07	–			
5. Negative Emotionality	−.03	−.58***	−.37***	−.54***	–		
6. IGD	.02	−.57***	−.19**	−.56***	.49***	–	
7. SMA	.10	−.53***	−.05	−.43***	.54***	.61***	–
*M*	39.66	37.38	36.36	41.27	38.70	19.08	16.25
*SD*	7.99	7.62	6.39	6.80	9.81	7.98	5.60
Actual range	20–60	21–56	16–60	22–58	13–59	9–41	6–30
Potential range	12–60	12–60	12–60	12–60	12–60	9–45	6–30
Cronbach’s α	.86	.85	.76	.78	.92	.92	.87
McDonald’s omega	.86	.86	.77	.80	.92	.93	.88

** *p* < .01. ***p < .001.

A series of standard multiple regressions was conducted to examine the relationships between the Big Five traits and IGD and SMA. The results are presented in Table 3. The predictors explained 41.3% of the variance in IGD, *F*(5, 240) = 33.70, *p* < .001. Conscientiousness (*beta* = −.30, *p* < .001) was the most important predictor followed by agreeableness (*beta* = −.29, *p* < .001) and negative emotionality (*beta* = .14, *p* = .037). The predictors explained 39.3% of the variance in SMA, *F*(5, 240) = 31.02, *p* < .001. Negative emotionality (*beta* = .41, *p* < .001) was the most important predictor followed by conscientiousness (*beta* = −.30, *p* < .001) and extraversion (*beta* = .16, *p* = .006).

**Table 3.** Standard Multiple Regression with Big Five Traits as the Predictors, and Internet Gaming Disorder (IGD) and Social Media Addiction (SMA) as the Criterion Variables.

Variables	*B*	*SE*	95% CI		β	*t*	*p*
			*LL*	*UL*			
IGD							
Open-mindedness	0.01	0.05	−0.01	0.11	.01	0.11	.914
Conscientiousness	−0.31	0.07	−0.45	−0.17	−.30	−4.39	**<.001**
Extraversion	−0.06	0.07	−0.20	0.08	−.05	−0.86	.391
Agreeableness	−0.34	0.08	−0.50	−0.19	−.29	−4.41	**<.001**
Negative emotionality	0.12	0.06	0.01	0.22	.14	2.10	.**037**
SMA							
Open-mindedness	0.03	0.04	−0.04	0.11	.05	0.93	.355
Conscientiousness	−0.22	0.05	−0.32	−0.12	−.30	−4.28	**<.001**
Extraversion	0.14	0.05	0.04	0.24	.16	2.80	.**006**
Agreeableness	−0.04	0.06	−0.14	0.08	−.04	−0.63	.532
Negative emotionality	0.23	0.04	0.16	0.31	.41	5.94	**<.001**

*Note.* Significant *p* values are bolded. CI = confidence interval; *LL* = lower limit; *UL* = upper limit.

### The Big Five Facets

A series of Pearson correlations were conducted to examine the relationships between the Big Five facets and IGD and SMA. The results are presented in Table 4. Organization (C), productiveness (C), responsibility (C), energy level (E), assertiveness (E), compassion (A), respectfulness (A), and trust (A) were negatively correlated with IGD whereas anxiety (N), depression (N), and emotional volatility (N) were positively correlated with IGD. Organization (C), productiveness (C), responsibility (C), assertiveness (E), compassion (A), respectfulness (A), and trust (A) were negatively correlated with SMA whereas sociability (E), anxiety (N), depression (N), and emotional volatility (N) were positively correlated with SMA.

**Table 4.** Descriptives and Pearson Correlations of the Big Five Facets, Internet Gaming Disorder (IGD), and Social Media Addiction (SMA).

Variables	1	2	3	4	5	6	7	8	9	10	11	12	13	14	15	16	17
1. IC (O)	–																
2. AS (O)	.67***	–															
3. CI (O)	.65***	.53***	–														
4. Org (C)	−.18**	−.10	−.15*	–													
5. Prod (C)	−.08	−.02	.04	.61***	–												
6. Res (C)	−.07	.09	.07	.46***	.68***	–											
7. Soci (E)	.09	−.07	.14*	−.10	.08	.03	–										
8. EL (E)	.25***	.15*	.33***	.02	.24***	.23***	.46***	–									
9. Assert (E)	.18**	.17**	.31***	.14*	.34***	.42***	.33***	.30***	–								
10. Com (A)	−.06	.02	.01	.38***	.36***	.35***	−.13*	.15*	.01	–							
11. Resp (A)	−.02	.00	.02	.45***	.50***	.50***	−.14*	.15*	.04	.59***	–						
12. Trust (A)	−.11	.06	−.06	.42***	.46***	.48***	−.02	.18**	.19**	.47***	.63***	–					
13. Anx (N)	.10	−.02	−.08	−.20**	−.46***	−.56***	−.18**	−.22***	−.43***	−.09	−.31***	−.40***	–				
14. Dep (N)	.05	−.06	−.10	−.32***	−.58***	−.64***	−.15*	−.33***	−.45***	−.29***	−.51***	−.57***	.75***	–			
15. EV (N)	.03	−.08	−.05	−.28***	−.51***	−.56***	.02	−.22**	−.37***	−.25***	−.56***	−.61***	.67***	.79***	–		
16. IGD	.07	−.03	−.01	−.41***	−.53***	−.53***	−.04	−.17**	−.24***	−.33***	−.53***	−.53***	.33***	.51***	.49***	–	
17. SMA	.11	.10	.03	−.43***	−.49***	−.42***	.13*	−.08	−.18**	−.22**	−.44***	−.42***	.40***	.52***	.55***	.61***	–
*M*	13.89	12.45	13.33	13.34	12.06	11.98	12.20	12.68	11.48	14.33	14.54	12.39	14.13	12.02	12.55	19.08	16.25
*SD*	3.08	3.55	2.62	3.22	3.07	2.67	3.03	2.50	2.89	2.39	2.72	2.96	3.34	3.77	3.69	7.98	5.60
Actual Range	5–20	4–20	8–20	6–20	5–19	5–19	4–20	4–20	4–20	8–20	7–20	4–20	5–20	4–20	4–20	9–41	6–30
Potential Range	4–20	4–20	4–20	4–20	4–20	4–20	4–20	4–20	4–20	4–20	4–20	4–20	4–20	4–20	4–20	9–45	6–30
Cronbach’s alpha	.69	.75	.61	.73	.69	.65	.70	.54	.65	.36	.63	.58	.79	.83	.83	.92	.87
McDonald’s omega	.70	.77	.60	.75	.70	.64	.71	.56	.66	.34	.54	.67	.79	.84	.83	.93	.88

*Note.* O = open-mindedness; C = conscientiousness; E = extraversion; A = agreeableness; N = negative emotionality; IC = intellectual curiosity; AS = aesthetic sensitivity; CI = creative imagination; Org = organization; Prod = productiveness; Res = responsibility; Soci = sociability; EL = energy level; Assert = assertiveness; Com = compassion; Resp = respectfulness; Anx = anxiety; Dep = depression; EV = emotional volatility.

* *p* < .05. **p < .01. ***p < .001.

A series of standard multiple regressions was conducted to examine the relationships between the Big Five facets and IGD and SMA. Because energy level (E) and compassion (A) were unreliable (both Cronbach’s alpha and McDonald’s omega < .60), these facets were omitted from the analyses (i.e., 13 facets instead of 15 facets as predictors). The results are presented in Table 5. The predictors explained 44.2% of the variance in IGD, *F*(13, 232) = 14.13, *p* < .001. Trust (A) (*beta* = −.17, *p* = .020) was the most important predictor followed by respectfulness (A) (*beta* = −.17, *p* = .027) and responsibility (C) (*beta* = −.17, *p* = .032). The predictors explained 43.8% of the variance in SMA, *F*(13, 232) = 13.93, *p* < .001. Emotional volatility (N) (*beta* = .29, *p* = .002) was the most important predictor followed by depression (N) (*beta* = .22, *p* = .033), organization (C) (*beta* = −.20, *p* = .003), aesthetic sensitivity (O) (*beta* = .18, *p* = .011), and sociability (E) (*beta* = .16, *p* = .006).

**Table 5.** Standard Multiple Regression with Big Five Facets as the Predictors, and Internet Gaming Disorder (IGD) and Social Media Addiction (SMA) as the Criterion Variables.

Variables	*B*	*SE*	95% CI		β	*t*	*p*
			*LL*	*UL*			
IGD							
Intellectual curiosity (O)	0.15	0.20	−0.26	0.55	.06	0.71	.477
Aesthetic sensitivity (O)	−0.07	0.16	−0.39	0.24	−.03	−0.46	.648
Creative imagination (O)	0.00	0.21	−0.42	0.42	.00	0.01	.995
Organization (C)	−0.13	0.17	−0.46	0.20	−.05	−0.76	.447
Productiveness (C)	−0.38	0.21	−0.79	0.03	−.15	−1.83	.068
Responsibility (C)	−0.51	0.24	−0.98	−0.05	−.17	−2.16	.**032**
Sociability (E)	−0.16	0.15	−0.4	0.14	−.06	−1.04	.297
Assertiveness (E)	−0.07	0.18	−0.43	0.29	−.03	−0.37	.709
Respectfulness (A)	−0.50	0.23	−0.94	−0.06	−.17	−2.22	.**027**
Trust (A)	−0.46	0.20	−0.85	−0.07	−.17	−2.35	.**020**
Anxiety (N)	−0.34	0.19	−0.72	0.04	−.14	−1.77	.078
Depression (N)	0.26	0.21	−0.16	0.68	.12	1.22	.223
Emotional volatility (N)	0.20	0.20	−0.19	0.59	.09	1.02	.308
SMA							
Intellectual curiosity (O)	−0.13	0.14	−0.41	0.16	−.07	−0.88	.382
Aesthetic sensitivity (O)	0.29	0.11	0.07	0.51	.18	2.57	.011
Creative imagination (O)	−0.10	0.15	−0.39	0.20	−.05	−0.66	.510
Organization (C)	−0.35	0.12	−0.59	−0.12	−.20	−3.01	.**003**
Productiveness (C)	−0.24	0.15	−0.52	0.05	−.13	−1.6	.109
Responsibility (C)	0.06	0.17	−0.27	0.39	.03	0.37	.709
Sociability (E)	0.29	0.11	0.08	0.50	.16	2.75	.**006**
Assertiveness (E)	0.07	0.13	−0.18	0.33	.04	0.57	.567
Respectfulness (A)	−0.00	0.16	−0.31	0.31	−.00	−0.01	.993
Trust (A)	−0.01	0.14	−0.29	0.26	−.01	−0.10	.923
Anxiety (N)	0.01	0.14	−0.26	0.28	.01	0.06	.956
Depression (N)	0.32	0.15	0.03	0.61	.22	2.14	.**033**
Emotional volatility (N)	0.44	0.14	0.17	0.72	.29	3.19	.**002**

*Note.* Energy level (E) and compassion (A) were omitted from the analyses because they were unreliable (both Cronbach’s alpha and McDonald’s omega < .60). Significant *p* values are bolded. CI = confidence interval; *LL* = lower limit; *UL* = upper limit.

For the sake of being thorough, a series of standard multiple regressions was conducted again with all 15 facets as predictors in an exploratory analysis. The predictors explained 44.4% of the variance in IGD, *F*(15, 230) = 12.24, *p* < .001. Respectfulness (A) (*beta* = −.19, *p* = .020) was the most important predictor followed by trust (A) (*beta* = −.18, *p* = .015) and responsibility (C) (*beta* = −.18, *p* = .028). The predictors explained 44.2% of the variance in SMA, *F*(15, 230) = 12.16, *p* < .001. Emotional volatility (N) (*beta* = .28, *p* = .003) was the most important predictor followed by depression (N) (*beta* = .23, *p* = .028), organization (C) (*beta* = −.21, *p* = .002), aesthetic sensitivity (O) (*beta* = .18, *p* = .013), and sociability (E) (*beta* = .16, *p* = .011).

## Discussion

The current study aimed to extend on a previous study (Wartberg et al., 2023) by examining the relationships between the Big Five traits and facets, and IGD and SMA among adults in the general population. Consistent with previous meta-analyses (Chew, 2022; Huang, 2022), the results supported the hypothesis that IGD would be negatively correlated with conscientiousness, extraversion, and agreeableness, and positively correlated with negative emotionality. The results also supported the hypothesis that SMA would be negatively correlated with conscientiousness and agreeableness, and positively correlated with negative emotionality. Furthermore, multivariate analyses showed that conscientiousness, agreeableness, and negative emotionality were significant predictors of IGD whereas negative emotionality, conscientiousness, and extraversion were significant predictors of SMA.

Conscientiousness appeared to be a protective factor for IGD and SMA. Highly conscientious individuals tend to be persistent, motivated, and prioritize goals in various life domains (e.g., school, work, or family) (Soto & John, 2017). Consequently, they are less likely to engage in problematic gaming or social media behaviors that could derail their goal-directed efforts. In contrast, negative emotionality appeared to be a risk factor for IGD and SMA. Individuals who are high on negative emotionality tend to be sensitive and more likely to experience negative emotions (Soto & John, 2017). Consequently, they might engage in games or social media to relieve or modify their negative moods (American Psychiatric Association, 2013; Griffiths, 2005). Unfortunately, this could result in a vicious cycle. For example, the use of social media could result in depression and anxiety due to negative interactions and social comparisons (Ivie et al., 2020; Seabrook et al., 2016; Yoon et al., 2019). The experience of negative moods might result in a greater need for social media for mood modification, leading to the maintenance or exacerbation of SMA.

With regards to the Big Five facets, the results showed that IGD was correlated with all facets except for intellectual curiosity (O), aesthetic sensitivity (O), creative imagination (O), and sociability (E). Similarly, SMA was correlated with all facets except for intellectual curiosity (O), aesthetic sensitivity (O), creative imagination (O), and energy level (E). This was largely inconsistent with the previous study that found significant correlations between IGD and all facets except for assertiveness (E), and between SMA and all facets except for aesthetic sensitivity (O), sociability (E), and assertiveness (E) (Wartberg et al., 2023). Furthermore, multivariate analyses with unreliable facets omitted showed that trust (A), respectfulness (A), and responsibility (C) were significant predictors of IGD whereas emotional volatility (N), depression (N), organization (C), aesthetic sensitivity (O), and sociability (E) were significant predictors of SMA. This was also inconsistent with the previous study that found significant multivariate relationships between IGD and aesthetic sensitivity (O), organization (C), productiveness (C), assertiveness (E), and anxiety (N), and between SMA and anxiety (N) (Wartberg et al., 2023). The differences in the results might be explained by the different demographics of the samples. Specifically, the previous study’s sample consisted of adolescents at risk for IGD and SMA whereas the current study’s sample consisted of adults in the general population. However, it is unclear if the differences are due to the age groups (adolescents vs. adults) or risk status (at risk vs. general population) or both.

Trust (A), respectfulness (A), and responsibility (C) appeared to be protective factors for IGD. Individuals who assume the best about people and their intentions (i.e., more trusting) might be more receptive to advice about playing games in moderation, which could reduce their risk of developing IGD. Furthermore, highly respectful and responsible individuals treat others with respect and can be counted on to complete their tasks (Soto & John, 2017). Consequently, they are unlikely to play games excessively, which could result in conflicts with others (i.e., not respectful) and problems in various life domains (i.e., not responsible) (American Psychiatric Association, 2013). Emotional volatility (N) and depression (N) appeared to be the main risk factors for SMA whereas organization (C) appeared to be a protective factor for SMA. Similar to the trait-level relationship between negative emotionality and SMA, individuals who are emotional and tends to feel depressed might use social media to modify their negative moods (Griffiths, 2005), leading to a vicious cycle that maintains both the negative moods and SMA. Finally, highly organized individuals prefer to keep their items neat, tidy, and in order (Soto & John, 2017). This could apply to both physical and digital items. Specifically, there are numerous social media platforms (e.g., Reddit, Facebook, Instagram), each with their own user profiles and login credentials, involving different methods of interactions (e.g., via text, images, videos). Furthermore, for images and videos, users might have to download additional applications for advanced editing features. To maintain order, highly organized individuals might restrict themselves to fewer platforms and methods of interactions, leading to a lower risk of SMA.

The use of facets as predictors has yielded three advantages. First, there is a clearer distinction between IGD and SMA. At the trait level, both forms of behavioral addictions are similar, with conscientiousness as a protective factor and negative emotionality as a risk factor. In contrast, at the facet level, negative emotionality’s facets are instrumental in predicting SMA but not IGD. Furthermore, different facets of conscientiousness serve as protective factors for IGD and SMA. Second, there is a better understanding of the contributors to IGD and SMA, which could inform preventive efforts. For example, preventive efforts to address IGD could include psychoeducation to inculcate respectfulness and responsibility. In addition, preventive efforts to address SMA could target individuals who are emotional or depressed but not those who are anxious. Finally, there is an increased nuance in comparisons between studies. For example, at the trait level, negative emotionality was the most important predictor of SMA in both the current study and the previous study (Wartberg et al., 2023). In other words, the results are consistent between studies. However, at the facet level, emotional volatility (N) and depression (N) were the two most important predictors for SMA in the current study whereas anxiety (N) was the only significant predictor for SMA in the previous study (Wartberg et al., 2023). The inconsistency between studies could provide greater insights into the condition and directions for future research (e.g., different risk factors for SMA for adolescents vs. adults).

Limitations of the study should be noted. First, two of the facets, energy level (E) and compassion (A), were unreliable. While facets tend to have lower reliability due to the smaller number of items (e.g., Soto & John, 2017), the Cronbach’s alpha and McDonald’s omega of the facets in the study was lower than expected. The facets were omitted from analyses and it is unclear how they would predict IGD and SMA if they were reliable. Furthermore, while respectfulness (A) and trust (A) were included in the analyses, it should be noted that their reliability varied as a function of the reliability test used (i.e., different result for Cronbach’s alpha vs. McDonald’s omega). Since both facets were significant predictors of IGD, the conclusions should be interpreted with caution. Second, the study used a cross-sectional design and the direction of causality is unclear. Given that personality traits are relatively stable over time and consistent across situations (Costa & McCrae, 1995), they were used as predictors in the current study. However, recent evidence suggested that traits are sufficiently malleable and could change in response to interventions (Bleidorn et al., 2018, 2021; Hudson & Fraley, 2015). For example, while it is possible that respectfulness (A) serves as a protective factor for IGD, it is also possible that prolonged engagement with problematic gaming could result in conflicts with others, leading to lower respectfulness (A). Finally, no covariates were used in the models because the focus of the study was to explore the use of the Big Five traits and facets as predictors of IGD and SMA. In the future these limitations might be controlled by using better instruments to assess the Big Five traits and facets, conducting longitudinal studies to assess the direction of causality, and including covariates in the models.

In conclusion, the findings of this study are important because it appears to be the first to examine the relationships between Big Five traits and facets, and IGD and SMA among adults in the general population. The results highlighted the advantages of using facets as predictors and have implications for both research and clinical practice. As more studies continue the trend of using both traits and facets, we can increase our understanding of the risk factors of IGD and SMA and inform preventive mental health strategies.
